# Brain Volume and Perception of Cognitive Impairment in People With Multiple Sclerosis and Their Caregivers

**DOI:** 10.3389/fneur.2021.636463

**Published:** 2021-05-06

**Authors:** Giuseppe Fenu, Lorena Lorefice, Elisa Carta, Mauro Arru, Alice Carta, Marzia Fronza, Giancarlo Coghe, Jessica Frau, Franco Contu, Maria Antonietta Barracciu, Eleonora Cocco

**Affiliations:** ^1^Multiple Sclerosis Center, Binaghi Hospital, Azienda Tutela della Salute (ATS) Sardegna, Cagliari, Italy; ^2^Multiple Sclerosis Center, Department of Medical Sciences and Public Health, University of Cagliari, Cagliari, Italy; ^3^Radiology Unit, Binaghi Hospital, Azienda Tutela della Salute (ATS) Sardegna, Cagliari, Italy

**Keywords:** multiple sclerosis, cognitive impairment, caregiver, brain volume, patients

## Abstract

**Background:** Cognitive impairment (CI) is common in people with multiple sclerosis (pwMS). The assessment of CI is based on neuropsychological tests and accurate anamnesis, involving the patients and caregivers (CG). This study aimed to assess the complex interplay between self-perception of CI, objective CI and the brain atrophy of MS patients, also exploring the possible differences with CI evaluated by caregivers.

**Methods:** Relapsing pwMS were enrolled in this study. Subjects underwent neuropsychological examination using the Brief Cognitive Assessment for Multiple Sclerosis (BICAMS) and evaluation of self-reported cognitive status using the patient-version of the Multiple Sclerosis Neuropsychological Questionnaire (p-MSNQ). Depression and anxiety were also evaluated using the Back Depression Inventory-version II (BDI-II) and Zung Anxiety Scale. Brain MRI images were acquired and brain volumes estimated. For each patient that was enrolled, we spoke to a caregiver and collected their perception of the patient's CI using the MSNQ- Caregiver version.

**Results:** Ninety-five MS subjects with their caregivers were enrolled. CI was detected in 51 (53.7%) patients. We found a significant correlation (*p* < 0.001) between BICAMS T scores and lower whole brain (Rho = 0.51), gray matter (Rho = 0.54), cortical gray matter (Rho = 0.51) volumes and lower p-MSNQ (Rho = 0.31), and cg-MSNQ (Rho = 0.41) scores. Multivariate logistic regression showed that p-MSNQ is related to a patient's anxiety to evaluate by Zung Score (*p* < 0.001) while cg-MSNQ to patient's brain volume (*p* = 0.01).

**Conclusion:** Our data confirm that neuropsychological evaluation results are related to the perception of CI and brain volume measures and highlight the importance of the caregiver's perception for cognitive assessment of pwMS.

## Introduction

Cognitive dysfunctions are frequent and represent a major concern for people living with multiple sclerosis (pwMS). Several studies estimated that the prevalence of cognitive impairment (CI) among pwMS ranges between 40 and 70%, occurring in subjects with different clinical course and MS features, early as in more advanced stages of the disease (1). In the last few years, growing attention has been paid to the evaluation of CI in MS, also because of the impact of this invisible but heavy symptom on several aspects of patients' lives. For this reason, numerous neuropsychological assessments have been proposed, including rapid screen tools principally useful in a clinical setting and self-reported questionnaires aimed to evaluate the perception of patients' cognitive functioning (2). The Brief International Cognitive Assessment for Multiple Sclerosis (BICAMS) is used in clinical settings, due to its rapidity of administration and the evaluation of principle cognitive domains affected by MS (3–5). The Multiple Sclerosis Neuropsychological Questionnaire (MSNQ), including a patient and caregiver (CG) version, has emerged as the most used tool worldwide for evaluating the perception of patients' CI (6). The relationship between the objective and perceived CI is notoriously extremely complicated and is potentially influenced by MS-related structural brain damage (7–9) as well as several others factors (10, 11) among which are also mood disorders (7, 8). Based on these considerations, this study aims to evaluate the complex interplay between CI of pwMS and the perception of cognitive functioning reported by patients and their CG, also exploring the possible relationships with brain volume measurements.

## Methods

### Participants

#### Patient Recruitment

Consecutive relapsing remitting pwMS were enrolled at the Multiple Sclerosis Center of Binaghi Hospital, ATS Sardegna. Exclusion criteria were: (i) exposure to corticosteroid or occurrence of clinical relapse in the previous 30 days; (ii) change in disease modifying therapy in the previous 6 months; (iii) presence of other chronic comorbidities; (iv) use of drugs or substances with a psychotropic effect; (v) contraindications to underwent MRI; (vi) presence of a physical disability that did not allow the neuropsychological evaluation (i.e., blindness).

All included MS patients underwent a clinical, neuropsychological, and brain MRI examination in the same week. Demographics and clinical MS features [gender, age, education, disease duration, and level of disability, assessed by Expanded Disability Status Scale (EDSS) score] (12) were also collected.

#### Caregiver Recruitment

For each enrolled patient, a caregiver was included. Caregivers were classified based on the relationship with the patients. Thus, the CG version of MSNQ (13) was administrated to the participants to capture their views on the patient's cognitive functioning. Informed consent was obtained from all participants (pwMS and CG) included in the study, which was approved by the local ethics committee.

#### Neuropsychological Assessment

The cognitive functions of the included patients were evaluated using the Italian version of the BICAMS battery (5) with implemented normative values for the Italian population and corrections for sex, age, and years of education (14). The BICAMS battery includes the Symbol Digit Modalities Test (SDMT) for evaluating the information processing speed, the California Verbal Learning Test (CVLT-II) for evaluating verbal learning and memory, and the Brief Visual Memory Test (BVMT) for evaluating visual learning and memory (5). In our study, according to the Italian validation process of the BICAMS battery, we included the total number of correct responses in 90 seconds for SDMT, the total number of words recalled over five learning trials (Total Learning, TL) for CVLT-II, and total recall score across the three trials.

According to the authors' definition, each test was classified as altered if the T Score was below 35 points. Thus, the self-perception of the CI of the patients was evaluated using the p-version of MSNQ (13).

The T score of any BICAMS tests was reported for each included patient, the mean T score of all BICAMS tests and the sum of BICAMS tests scored below 35 T score (number of altered tests). Finally, depression and anxiety were evaluated using the BDI-II and the Zung Anxiety Scale (15, 16).

#### MRI Acquisition

Brain MRIs were acquired using a Magnetom Avanto Scanner (Siemens, Enlargen) at 1.5 T. The MRI protocol included the following sequence: 3D T1-Magnetization Prepared Rapid Acquisition Gradient-Echo (MPRAGE): echo time (TE): 2.37 ms; repetition time (TR): 1,730 ms; inversion time (TI): 1,050 ms; field of view (FOV): 244 mm; voxel size: 1 × 1 × 1 mm, (176 contiguous slices). A dual-echo, turbo spin-echo sequence (repetition time/echo time 1/echo time 2 5 2,075/30/90 ms, 256 3256 matrix, one signal average, 250-mm field of view, 50 contiguous 3-mm slices) yielding proton density–weighted and T2-weighted images oriented to exactly match the MPRAGE image acquisition. Brain parenchyma volumes were measured on T1W gradient echo images using the cross-sectional version of SIENA (structural image evaluation using normalization of atrophy) software, SIENAX (part of FSL 4.0: http://www.fmrib.ox.ac.uk/fsl/), and a previously described method to estimate the overall brain volume, normalized for head size. MRI analysis allowed us to obtain normalized whole brain volume (WB), normalized gray matter volume (GM), normalized white matter volume (WM), and normalized cortical gray matter volume (cGM). T1 hypo-intense lesion refilling was performed as previously described (17, 18). The radiologist was blinded to the results of the cognitive and neurological evaluation.

### Statistical Analysis

All statistical analyses were performed using SPSS for Mac version 20.0 (SPSS Inc., Chicago. IL, USA). First, descriptive analysis was performed. Next, we used the Shapiro-Wilks and Kolmogorov-Smirnov for testing the normality of variables. Based on normal distribution evaluation, we used a parametric or non-parametric test to evaluate the correlation between the variables evaluated. the relationship of BICAMS Tests Results with brain volumes was assessed by Pearson or Spearman test. Analogously, the relationship of p-MSNQ and cg-MSNQ scores with BICAMS Tests Results and brain measurements were evaluated. Thus, regression analyses were performed to evaluate which factors influence p-MSNQ and cg-MSNQ scores, included in each model as dependent variable, also controlling for BDI-II and Zung Anxiety scores. Moreover, we performed a collinearity diagnostic test regarding the linear regression. For all assays, the statistical significance was set at *P* < 0.05.

The results were filtered using the Benjamini-Hochberg procedure for FDR correction (FDR < 0.05). The test of the collinearity of variables also included multivariate linear regression analysis.

## Results

The sample included 95 MS relapsing remitting patients (68/95; 71.6% female). Mean values for age and disease duration were, respectively, 43.65 (SD: 11.9) and 12.1 (SD: 7.8) years, while the median EDSS score was 2.0 (IQR: 0–5.5). For each MS patient, a caregiver was included. Of these, 62 were partners (65.2%), and 33 family caregivers (34.8%). Table 1 shows the demographic and clinical features of participants included in the study. CI, defined by at least one impaired test at the BICAMS assessment, was relieved in 51 (53.7%) of patients.

**Table 1:** Demographic and clinical features of pwMS and their caregivers.

	**Pw MS (95)**	**CG (95)**
Female	68 (71.6%)	60 (63.1%)
Age (mean ± sd) years	43.65 ± 11.9	49.5 ± 10.2
Education (mean ± sd) years	13 ± 3.5	12.3 ± 4.4
MS duration (mean ± sd)	12.1 ± 7.8	
EDSS score Median (IQR)	2.0 (0–5.5)	
Whole Brain volume ml (mean ± sd)	1434.55 ± 99.68	
White matter ml (mean ± sd)	673.66 ± 37.30	
Gray matter ml (mean ± sd)	760.88 ± 78.58	
Cortex ml (mean ± sd)	594.51 ± 62.00	
SDMT T scores (mean ± sd)	42.02 ± 11.17	
CVLT T scores (mean ± sd)	44.30 ± 13.77	
BVMT T scores (mean ± sd)	48.11 ± 12.44	
BICAMS T scores (mean ± sd)	44.9 ± 10.57	

We found a significant correlation of mean BICAMS T scores with measurements of WB (Rho = 0.50), GM (Rho = 0.545), and cGM (Rho = 0.517), (*p* < 0.001), as shown in Table 2.

**Table 2:** Correlations of brain volume with T scores at BICAMS assessment.

	**N # failed**	**SDMT**	**CVLT**	**BVMT**	**BICAMS**
	**tests**	**T scores**	**T scores**	**T scores**	**Mean**
					**T scores**
Whole brain	−0.423^**^	0.495^**^	0.389^**^	0.456^**^	0.501^**^
White matter	−0.137	0.217^*^	0.157	0.181	0.186
Gray matter	−0.501^**^	0.523^**^	0.420^**^	0.494^**^	0.545^**^
Cortex	−0.478^**^	0.485^**^	0.410^**^	0.461^**^	0.517^**^

* *p < 0.01.*

** *p < 0.001*.

As shown in Table 3, the relationship of mean BICAMS T scores with p-MSNQ (Rho = 0.31 *p* < 0.01) and cg-MSNQ (Rho = 0.41; *p* < 0.001) is also observed. In addition, the perception of CG, as indicated by cg-MSNQ score, inversely correlates with WB (Rho = −0.495), GM (Rho = −0.554) and cGM (Rho = −0.563) volumes. No significant correlation was found between the patient's point of view, indicated as p-MSNQ scores, and brain volume measurements (Table 3).

**Table 3:** Correlations of p-MSNQ and cg-MSNQ scores with BICAMS results and brain volume measurements.

	**p-MSNQ scores**	**cg-MSNQ scores**
N# failed tests	0.168	0.401^**^
SDMT T scores	−0.349^**^	−0.451^**^
CVLT T scores	−0.300^**^	−0.328^**^
BVMT T scores	−0.217^*^	−0.328^**^
BICAMS T scores	−0.317^*^	−0.416^**^
Whole brain	−0.131	−0.495^**^
White matter	−0.197	−0.116
Gray matter	−0.072	−0.554^**^
Cortex	−0.004	−0.563^**^
Zung scores	0.593^**^	0.232
BDI scores	0.225	0.008

* *p < 0.01.*

** *p < 0.001*.

A multivariate linear regression model was also performed. First, we included as dependent variable p-MSNQ founding a significant association of p-MSNQ scores with anxiety evaluated by Zung scores (*P* = 0.001) also controlling for BDI results, mean of BICAMS T scores, and brain volume (Table 4A). Moreover, we performed another analysis including the cg-MSNQ score as a dependent variable, highlighting a relationship with the patients' lower brain volume (*p* = 0.01) with no significant relationship with depression, anxiety, and BICAMS results (Table 4B). The variance inflation factor (VIF) values and the condition index results were not indicative of collinearity for variables included in multivariate linear regression analysis.

**Table 4:** Multiple regression analyses.

**A: Dependent Variable: p-MSNQ Scores**			**B: Dependent Variable: cg-MSNQ Scores**		
**Independent variables**	**Standardized beta**	***p*-value**	**Independent variable**	**Standardized beta**	***p*-value**
Bdi scores	−0.100	ns	BDI scores	−0.060	ns
Zung scores	0.622	**0.001**	Zung scores	0.129	ns
Bicams mean T score	0.087	ns	BICAMS mean T score	−0.203	ns
Whole brain	−0.191	ns	Whole brain	−0.429	**0.01**

*Multiple linear regression analysis was used to examine the relationship between p-MSNQ and cg-MSNQ scores, included in the model as a dependent variable, with BDI-II, Zung, BICAMS T scores, and whole brain volume (independent variables).*

*Relationship of the number of p-MSNQ and cg-MSNQ scores with depression, anxiety, mean BICAMS T scores, and brain volume measurements. Bold values are statistically significant with a p < 0.05*.

## Discussion

Our results confirmed the universally recognized role of MRI analysis as principal biomarkers of cognitive functions in MS (19). The present study also found a strong correlation between the volumes of the whole brain, gray matter, and cortical volume with the results of cognitive tests.

As observed in other neurological diseases, MRI measurements are not enough to fully explain cognitive deficits in MS (20). In recent decades several studies have aimed to investigate how other factors play a role (21). Among these factors, cognitive reserve, several demographic, clinical, mood disorders, and social variables could act as moderators (22, 23). However, brain volume measures showed a strong and significant relationship with all cognitive functions evaluated and the global cognitive status of MS patients.

The other aim of our study was to explore the reliability perception of cognitive impairment in Multiple Sclerosis. The data show that caregiver perception is more strongly correlated to the objective cognitive performance of people with MS than their self-judgment. In other neurological pathologies such as neurodegenerative diseases, it is a common observation that the cognitive ability self-perception of the patient is less accurate than caregiver perception (24–26).

As previously described, cognition self-judgment is often more conditioned by mood disorders such as depression and anxiety than by objective cognitive deficit (27). A severe mood disorder could interfere with both anamnestic interview and neuropsychological evaluation (28), complicating the estimation of cognitive functions and leading to overestimation of the impairment of cognitive abilities. As in our cohort, the perception of cognitive functioning reported by patients appeared to be related to anxiety in a model controlled for brain volume and the results of neuropsychological assessment (28).

Several other previous studies have evaluated the reliability of cognitive function self-judgment compared to caregiver evaluation and relationship with a mood disorder. O'Brien et al. found that p-MSNQ correlated with depression as assessed by BDI, while cg-MSNQ was independent from mood disorders, but was correlated with cognitive impairment as assessed by an extended neuropsychological battery (29). Another previous study indicated that in MS patients, after controlling for demographic variables, anxiety was a significant predictor of p-MSNQ scores, while the patients' point of view did not correlate with the results of neuropsychological examination (30). A recent study, conducted on the Danish MS population confirmed that the p-MSNQ version measures these items more than the cognitive abilities of the patients (31). These previous studies are in line with our results which confirm that the patient's self-assessment of their cognitive functions is related more to the characteristics of their mood than to objective evaluation.

Interestingly, the relationship between caregiver perception of a patient's cognition and patients' brain volume emerged as an unexpected result of our study. The perception of CI reported by the caregivers shows a strong correlation with patient brain volume measures, whole brain, and gray matter, while there is no correlation between p-MSNQ and brain atrophy. In the multivariate analysis, the cg-MSNQ scores were also related to patients' brain volume, even after controlling for depression BDI-II scores, anxiety Zung scores, and neuropsychological test results. As previously described (28), the caregiver's evaluation of the patient's cognitive functions is based on multiple issues such as skills in daily life, detailed knowledge of the premorbid level of cognitive skills, and the social context of the patient. Consequently, our data support the hypothesis that the perception of the caregiver is related to the effective cognitive functioning of the patient as documented by the strong correlation with the brain volume confirmed also in the multivariate analysis. Thus, caregiver evaluation of cognitive functioning in MS emerges as related to brain volume as an indication of structural damage. The absence of a correlation between patient self-evaluation and brain volume measure could be explained by processes such as the influence of mood disorders, especially anxiety, on self-evaluation and a lack of insight about impairment in patients with severe brain atrophy.

Recently, several studies on metacognition have also contributed to the understanding of the complex interplay that regulates the perception of cognitive disorders in MS (32). These findings are in line with our results and point to the role of mood disorders in self-perception of cognitive impairment in people with MS. Our study also adds the significant relationship between the caregiver's point of view, cognitive measures and brain volume as the main biomarker of cognitive impairment.

Our study shows several limitations. First, the limited number of pwMS included in the research could influence the application of the results. Second, the MRI biomarkers included only the brain volume measurements while also other radiological features are associated with CI in MS as white matter total lesion load that was not included in the present study. Furthermore, even if using appropriate statistical tests, given the limited size of the sample, it is not possible to exclude errors due to the association between the evaluated measures.

## Conclusions

In conclusion, our study confirmed the well-known importance of MRI volumetric measurements as biomarkers of CI in MS based on the relationship with cognitive results. Furthermore, the caregiver's point of view appears to be stronger related to neuroradiological biomarkers of cognitive deficit and neuropsychological assessment test results rather than patient self-evaluation.

This data suggests the importance of including the caregivers' judgment in the anamnestic evaluation of pwMS undergoing neuropsychological assessment. Further studies are needed to better evaluate what tools to use in a clinical setting to capture both MS patients' and caregivers' perceptions.

## Data Availability Statement

The raw data supporting the conclusions of this article will be made available by the authors, without undue reservation.

## Ethics Statement

The studies involving human participants were reviewed and approved by University of Cagliari. The patients/participants provided their written informed consent to participate in this study.

## Author Contributions

GF and LL participated in the design of the study and drafted the manuscript. ECa, MA, MF, and AC carried out the neuropsychological evaluation and performed the statistical analysis and drafted the manuscript. JF and GC revised the manuscript for important intellectual content and performed the statistical analysis. FC and MB acquired MRI images. ECo helped draft the manuscript and revised it critically for important intellectual content. All authors read and approved the final manuscript.

## Conflict of Interest

GF was an editorial board member of BMC Neurology and received a travel grant, speaker fee, and consultancy from Biogen Idec, Sanofi, Teva, Admirall, Genzyme, Merck Serono, and Novartis. LL, GC, JF, and ECo received travel grants, speaker fees, and consultancy from Biogen Idec, Sanofi, Teva, Admirall, Genzyme, Merck Serono, and Novartis. MB and FC received travel grants from Biogen Idec. The remaining authors declare that the research was conducted in the absence of any commercial or financial relationships that could be construed as a potential conflict of interest.

## Abbreviations

BICAMS: Brief International Cognitive Assessment for Multiple Sclerosis
BVMT: Brief Visual Memory Test-Revised
CFs: Cognitive Functions
cgMSNQ: Multiple Sclerosis Neuropsychological Questionnaire- caregiver version
CGs: Caregivers
CI: Cognitive impairment
CVLT: California Verbal Learning Test
MS: Multiple Sclerosis
pMSNQ: Multiple Sclerosis Neuropsychological Questionnaire-patient version
SDMT: Symbol Digit Modalities Test
WB: whole brain
WM: whole white matter
GM: whole gray matter
cGM: cortical gray matter.
